# SDF-1α mRNA therapy in peripheral artery disease

**DOI:** 10.1007/s10456-025-09979-3

**Published:** 2025-05-02

**Authors:** Tinghong Zhang, Binqiang Zhu, Shijie Deng, Jinling Qin, Jingyuan Zhang, Shu Meng

**Affiliations:** 1 State Key Laboratory of Respiratory Disease, the First Affiliated Hospital, Guangzhou Medical University, Guangzhou, Guangdong 510120 China; 2 Department of Basic Science Research, Guangzhou National Laboratory, Guangzhou, Guangdong 510005 China

**Keywords:** SDF-1α, mRNA therapy, Angiogenesis, Arteriogenesis, Ischemic disease

## Abstract

Stromal cell-derived factor-1 alpha (SDF-1α) is a promising target for therapeutic angiogenesis in ischemic diseases such as peripheral artery disease (PAD). However, the clinical application of SDF-1α protein or plasmid-based gene therapy is unsuccessful. mRNA therapy has shown great promise in protein replacement. Here, we developed DOPE-lipid nanoparticles (LNPs) encapsulating SDF-1α mRNA (LNP@SDF-1α) for efficient gene delivery. In vitro, LNP@SDF-1α treatment of human umbilical vein endothelial cells (HUVECs) significantly enhanced endothelial migration, tube formation, and increased monocyte adhesion, demonstrating robust pro-angiogenic activity. In vivo, LNP@SDF-1α transfected HUVECs showed enhanced angiogenic capacity in a murine Matrigel plug model. Furthermore, in a mouse hindlimb ischemia model, intramuscular injection of LNP@SDF-1α into ischemic limbs accelerated blood flow recovery, as assessed by laser speckle contrast imaging. Immunofluorescence staining revealed a marked increase in capillary and arteriole densities in treated tissues. Angiogenic protein profiling demonstrated an upregulation of pro-angiogenic factors, including VEGF and Ang-1, and a downregulation of anti-angiogenic factors. No significant toxicity was observed in major organs, indicating the safety of this approach. Our study demonstrates that SDF-1α mRNA therapy, delivered via DOPE-LNPs, significantly promotes vascular regeneration in ischemic tissues by enhancing angiogenesis and arteriogenesis, thereby restoring blood perfusion. This approach presents a promising therapeutic option for PAD and suggests broader applications of mRNA-based therapies for ischemic diseases.

**Supplementary Information:**

The online version contains supplementary material available at 10.1007/s10456-025-09979-3.

## Introduction

Peripheral artery disease (PAD) is the third leading cause of atherosclerotic cardiovascular morbidity, following coronary artery disease and stroke, affecting more than 200 million individuals worldwide [1, 2]. PAD primarily impacts the lower limbs, leading to ischemia, pain, and impaired mobility. While medical therapies that target cardiovascular risk factors have advanced, treatment options that directly enhance limb perfusion remain limited [3, 4]. Severe cases often necessitate surgical revascularization or endovascular interventions; however, these approaches are not universally effective or feasible [3, 5, 6]. Thus, there is an urgent need for novel therapeutic strategies, especially for advanced PAD.

Recent advances in regenerative medicine have highlighted the potential of vascular regeneration therapies to enhance tissue perfusion and improve outcomes in PAD [7]. Among these, stromal cell-derived factor-1 (SDF-1), also known as CXCL12, is a promising candidate for therapeutic vascular regeneration due to its role in mobilizing endothelial progenitor cells (EPCs) and enhancing neovascularization in ischemic tissues [8, 9]. By binding to the CXCR4 receptor, SDF-1 facilitates the mobilization and homing of EPCs to ischemic sites, thereby aiding in vascular regeneration [10, 11]. Although SDF-1 expression is naturally upregulated in response to ischemia, this endogenous increase is transient and often insufficient for full restoration of blood perfusion [12]. Preclinical studies have demonstrated that exogenous SDF-1 protein administration enhances angiogenesis and accelerates wound healing in PAD models [13, 14]. However, the short half-life and high costs of protein-based therapy have limited its clinical application. Similarly, DNA-based SDF-1 gene therapy has encountered substantial obstacles. Large randomized controlled trials using plasmid-based SDF-1 overexpression via intramuscular injection failed to demonstrate significant improvements in walking distance or amputation rates [15], likely due to low transfection efficiency and insufficient protein expression.

mRNA therapy has opened up new avenues for delivering therapeutic proteins. mRNA therapy offers several advantages over traditional protein or gene therapies, such as transient and controllable expression, reduced immunogenicity, and the potential for rapid clinical translation. Preclinical studies with mRNA platforms have shown encouraging results, improving blood flow and reducing tissue necrosis in hindlimb ischemia models that closely mimic human PAD [16]. We hypothesize that SDF-1α mRNA therapy can overcome the limitations of protein- and plasmid-based therapies by enabling efficient expression of SDF-1α, thus enhancing its regenerative potential in ischemic tissues.

In this study, we investigated the therapeutic efficacy of SDF-1α mRNA therapy both in primary endothelial cells (ECs) in vitro and murine models of angiogenesis in vivo. We developed a highly efficient lipid nanoparticle (LNP) delivery system for SDF-1α mRNA. In vitro, SDF-1α mRNA enhanced endothelial migration, tube formation, and CXCR4^+^ cell adhesion. In vivo, SDF-1α mRNA promoted angiogenesis in a Matrigel plug model and significantly improved blood flow recovery, vascular density, and functional outcomes in ischemic limbs, highlighting its potential as a novel therapeutic approach for PAD and broader ischemic diseases.

## Materials and methods

### Materials

Cholesterol and 1,2-dioleoyl-sn-glycero-3-phosphoethanolamine (DOPE), DSPC (1,2-Distearoyl-sn-glycero-3-phosphorylcholine) was from Avanti Polar Lipids. DMG-PEG 2000 and Dlin-MC3-DMA was from Sigma-Aldrich and MedChemExpress, respectively. Quant-iT RiboGreen, Lipofectamine™ 3000 and SDF-1α (PA5-114344) antibody was from Thermo Fisher Scientific. Pseudouridine, 5-methylcytosine, synthesized mRNA of mCherry and firefly luciferase with N1-Methyl-Pseudouridine and 5-methylcytosine substitutions were from Apexbio. FastPure cell/tissue total RNA isolation kit and HiScriptIII RT SuperMix for qPCR Kit were from Vazyme. Human umbilical vein ECs (HUVECs), EBM2 medium, and EGM2 bullet kit were purchased from Lonza. Cell Counting Kit-8 (CCK-8) was from Dojindo. Recombinant human SDF-1α protein was from Sino Biological Inc. Matrigel was from Corning. CD31 (ab182981) and α-SMA (ab7817) antibodies and human SDF1 ELISA kit (ab100637) were purchased from Abcam. Proteome Profiler Mouse Angiogenesis Array Kit was from R&D Systems. AMD3100 8HCl (S3013) was obtained from Selleck Chemicals. T7 High Yield RNA Synthesis Kit and Monarch^®^ RNA cleanup kit were purchased from New England Biolabs. DNase I was obtained from Promega Corporation.

### mRNA synthesis and purification

Modified linear SDF-1α mRNA contains a human SDF-1α coding region, 5’ and 3’ untranslated region that was derived from human β-globin, a cap 1 structure, and a 120-nucleotide poly A tail. In brief, mRNA was synthesized in vitro by T7 RNA polymerase mediated transcription from a linearized DNA template with complete replacement of uridine and cytosine along the entire mRNA with pseudouridine and 5-methylcytosine, respectively. After in vitro transcription, the productions were treated with DNase I for 15 min. After DNase treatment, linear mRNA was purified using a Monarch^®^ RNA cleanup kit.

### Synthesis and characterization of nanoparticles

To prepare LNPs@mRNA, the ethanol phase consisted of a mixture of Dlin-MC3-DMA, cholesterol, DOPE or DSPC, and DMG-PEG 2000 at varying mole ratios. The aqueous phase was prepared using 10 mM citrate buffer (pH 3.0) containing modified mRNA. LNPs@mRNA were synthesized by mixing the aqueous and ethanol phases at a 3:1 ratio, with a final mRNA concentration of 0.1 mg/mL. The mass ratio of Dlin-MC3-DMA to mRNA was 10:1. The resulting LNPs (LNP@SDF-1α or LNP@mCherry) were dialyzed in 1× PBS using a 20,000 MWCO cassette at 4 °C for 3 h.

Encapsulation efficiency was measured with Quant-iT RiboGreen. Briefly, LNP@mRNA was treated with TE buffer (for unencapsulated mRNA) or TE buffer with 1% Triton X-100 (for total mRNA). Fluorescence was measured after adding RiboGreen reagent, and RNA content was quantified using a standard curve to calculate encapsulation. Encapsulation efficiency (EE%) was calculated as follows:

$$\:EE\%=\left(\frac{1-unencapsulated\:mRNA}{total\:mRNA}\right)\times\:100\%$$

Z-average diameter, polydispersity index (PDI), and zeta potential of nanoparticles were analyzed using a Zetasizer Nano ZS (Malvern Instruments, U.K.) [17]. For transmission electron microscopy (TEM), 3 µL LNP solution was placed on a carbon-coated copper grid and stained with 2% uranyl acetate for 1 min. Images were captured using an FEI Talos F200S microscope (Hillsboro, OR, U.S.A).

### SDF-1α plasmid construction

The human SDF-1α gene was amplified from human umbilical vein endothelial cells (HUVECs) -derived cDNA by polymerase chain reaction (PCR) using specific primers (forward: 5’-CGGAATTCATGAACGCCAAGGTCGTG-3’, reverse 5’-CGGAATTCATGAACGCCAAGGTCGTG-3’), designed to introduce EcoRI and XhoI restriction sites to the 5’ and 3’ ends, respectively. The resulting PCR products and the backbone plasmid pcDNA3.1(+) were digested separately with EcoRI and XhoI restriction enzymes. Following purification, the digested products were ligated using T4 DNA ligase, generating the recombinant plasmid pcDNA-SDF-1α.

### Cell culture

HUVECs were cultured in EGM2 medium. The medium was changed every other day [18]. Human acute monocytic leukemia cell line THP-1 was cultured in RPMI-1640 medium supplemented with 10% FBS. All cells were maintained at 37 °C in a 5% CO_2_ humidified atmosphere.

### Cytotoxicity assay

LNP cytotoxicity was assessed by treating HUVEC monolayers with LNPs at different concentrations (12.5, 25, 50, and 100 µg/mL). Control cells received medium alone. After 48–72 h, 100 µL fresh medium with 10 µL CCK-8 reagent was added to the cells for 4 h. Absorbance at 450 nm was measured using a microplate reader (Cytation 5, BioTek Instruments, Inc. GA, U.S.A), and the ratio of absorbance between LNP-treated and control cells was calculated [19].

### Flow cytometry (FACS) analysis

To assess cellular uptake of LNP@mRNA, HUVEC monolayers were treated with LNP@mCherry (1 µg mCherry mRNA) for 6, 18, or 36 h. Free mCherry mRNA served as a control. Cells were harvested by trypsinization. Mean fluorescence intensity (MFI) and percentage of mCherry-positive cells were determined by flow cytometry using a CytoFLEX machine (Beckman Coulter, CA, U.S.A).

### RNA extraction and Real-Time PCR (RT-PCR)

Total RNA was extracted from HUVECs or tissues and reverse transcribed into cDNA. Real-time PCR was performed using SYBR Green master mix on a QuantStudio 3 machine (Thermo Fisher Scientific). Gene expression was analyzed using the ΔΔCt method, normalized to *β-ACTIN* (human) or *Gapdh* (mouse). Primer sequences for specific genes are listed as follows: mouse *Gapdh*: forward 5’-CATCACTGCCACCCAGAAGACTG-3’ and reverse 5’-ATGCCAGTGAGCTTCCCGTTCAG-3’ *β-ACTIN*, forward 5’-GTGAAGGTGACAGCAGTCGGTT-3’ and reverse 5’-GAAGT GGGGTGGCTTTTAGGA-3’, human *SDF-1α*, forward 5’-AGATGCCCATGCCGATTCTTCG-3’ and reverse 5’-CGGGTCAATGCACACTTGTCTG-3’ *Hif-1α*, forward 5’-ACCTTCATCGGAAACTCCAAAG-3’ and reverse 5’-ACTGTTAGGCTCAGGTGAACT-3’ *Vegf-a*, forward 5’-CTGCTGTAACGATGAAGCCCTG-3’ and reverse 5’-GCTGTAGGAAGCTCATCTCTCC-3’.

### Tube formation assay

Angiogenesis was assessed via a tube formation assay. 24-well plates were coated with 300 µL Matrigel and incubated at 37 °C for 30 min. HUVECs were treated with LNP or LNP@SDF-1α for 6 h, then the treated cells were plated in the 24-well plates (7 × 10^4^ cells/well). After 16 h, cells were stained with CellTracker Red (5 µM) for 30 min and visualized under a fluorescence microscope. The total vessel length was quantified using AngioTool (NCI) [20].

### Monocyte-EC adhesion

HUVECs were plated in 12-well plates and allowed to form a confluent monolayer. Cells were treated with LNP-delivered SDF-1α for 6 h. THP-1 monocytes were loaded with CellTracker Red (8 µM) for 30 min. Labeled THP-1 cells (1 × 10^5^) were added to the ECs and incubated at 37 °C for 30 min. Non-adherent cells were washed away with PBS, and images were taken using EVOS, with quantification performed using ImageJ.

For the adhesion inhibition assay, HUVECs were treated with LNP-delivered SDF-1α 0.4 µg for 6 h. THP-1 cells were pre-incubated with 10 µM CXCR4 antagonist (AMD3100 8HCl) for 30 min. Then, the pre-treated THP-1 cells were added to the ECs in EGM2 medium containing 10 µM AMD3100 8HCl for 30 min.

### Migration wound-healing assay

HUVECs were seeded into 6-well plates, and at 90% confluence, the cells were treated with SDF-1α mRNA-loaded LNPs for 6 h. A scratch was made using a sterile pipette tip. After washing with PBS, cells were incubated in EGM-2 medium, and the wound area was monitored under a phase-contrast microscope (Leica, Germany). Wound closure was quantified using ImageJ.

### ELISA assay

HUVECs (4 × 10⁵) were seeded into 6-well plates and transfected with either LNP-encapsulated SDF-1α mRNA (1 µg) or an SDF-1α plasmid (1 µg) using Lipofectamine™ 3000. After 16 h, the culture supernatant was collected, and SDF-1α levels were quantified using an ELISA assay kit according to the manufacturer’s instructions.

### Animal care and use

All relevant procedures involving animal experiments presented in this study are compliant with ethical regulations regarding animal research and were conducted under the approval of the Animal Care and Use Committee of the Guangzhou National Laboratory.

### Matrigel plug assay

HUVECs were transfected with LNP or LNP@SDF-1α for 6 h. Then 1 × 10^6^ treated cells were mixed with 300 µL Matrigel containing VEGF (100 ng/mL) and heparin (60 IU/mL). These mixtures were injected subcutaneously into 8-week-old C57BL/6 mice. Matrigel plugs were removed after 5 days for histology and immunofluorescence staining.

### Murine hindlimb ischemia

Hindlimb ischemia was induced in 8-week-old male BALB/c mice as previously reported [18]. Briefly, unilateral hindlimb ischemia was induced by ligating the proximal end of the femoral artery. Afterwards, the mice were housed individually and blood flow in the affected and control limbs was imaged by up to 21 days. A total of 100 µL of LNP-encapsulated SDF-1α mRNA (20 µg) was injected into four sites of the gastrocnemius muscle (approximately 25 µL per site) immediately after surgery and again 3 days post-surgery. Blood perfusion was monitored immediately after surgery and 3, 7, 14, and 21 days postoperatively under Laser Speckle Contrast Imaging System (Rayward Life Technology, Shenzhen, China). The limb loss score was graded as autoamputation (Loss of Limb) (5), leg necrosis (up to the gastrocnemius muscle) (4), foot necrosis (3), toe necrosis (2), nail necrosis (1), or no changes (0) [18]. The scoring criteria of limb salvage is as follows: no change (limb salvage), nail or toe necrosis (toe necrosis), foot necrosis (foot necrosis), leg or whole limb loss (limb loss) [21]. There were two independent observers, blinded to the group assignment. The gastrocnemius muscles were collected at indicated time from the operated limbs. Five male mice were used for each experimental group [18, 21].

### mRNA protein expression in vivo

LNP-encapsulated firefly luciferase (Fluc) mRNA or free Fluc mRNA (4 µg) was injected into the gastrocnemius muscle of 8-week-old C57BL/6 mice. Fluc expression was assessed using an IVIS imaging system.

For SDF-1α expression, 2 µg of recombinant SDF-1α protein, 20 µg of naked SDF-1α plasmid, or 20 µg of LNP-encapsulated SDF-1α mRNA was injected into the gastrocnemius muscle of 8-week-old C57BL/6 mice. SDF-1α protein levels were quantified by ELISA 4 h post-injection.

### Immunofluorescence staining

Gastrocnemius muscles were harvested 21 days post-surgery, fixed in 4% paraformaldehyde, and embedded in paraffin. The fixed muscles were subsequently sectioned at 4 μm, permeabilized in 0.1% Triton X-100, and blocked in PBS containing 5% donkey serum for 1 h at room temperature. The sections were subsequently stained with CD31 or α-SMA antibodies, followed by DAPI nuclear staining. Images were captured using an Olympus SLIDEVIEW TM VS200 microscope.

### Mouse angiogenesis array

Protein lysates collected from the gastrocnemius muscles 21 days post-surgery were analyzed using the Mouse Angiogenesis Protein Array according to the manufacturer’s instructions. Data were quantified using ImageJ.

### Data analysis

Statistical analysis was performed with Prism 7 software (GraphPad Software, Inc). Results were expressed as the mean ± SEM. Statistical comparisons between two groups were performed via Student t-test. And a one-way ANOVA test was used to analyze multiple groups. *P < 0.05 were considered statistically significant. **P < 0.01, ***P < 0.001.

## Results

### Synthesis and characterization of DOPE-containing LNPs for mRNA delivery

**Fig. 1:**
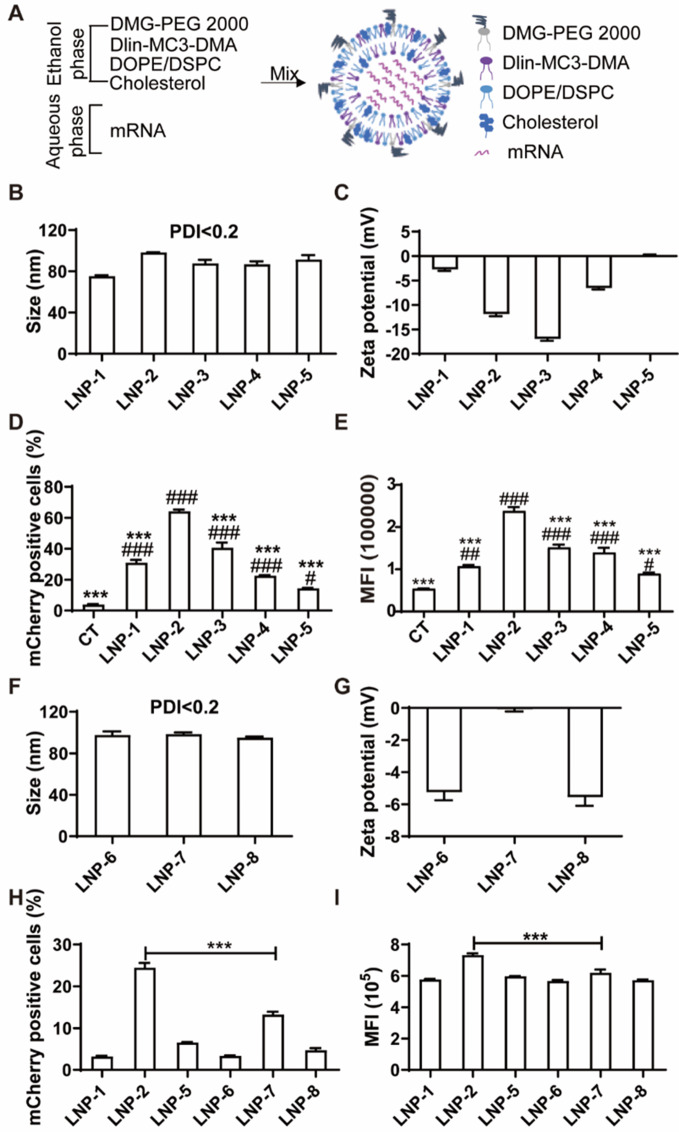
Characterization of LNPs with different phospholipids at different mole ratios and their delivery efficiency of mCherry mRNA in ECs. (**A**) Schematic of assembly of LNP. Sizes (**B**) and Zeta potentials (**C**) of DOPE containing LNPs at different mole ratios with mCherry mRNA. Quantification of mCherry positive cells (**D**) and Mean fluorescence intensity (**E**) of HUVECs transfected with DOPE-LNP encapsulated with mCherry mRNA at 24 h post-transfection. # *P* < 0.05, ## *p* < 0.01, ## *P* < 0.001 versus control; *** *P* < 0.001 versus LNP-2 group. Sizes (**F**) and zeta potentials (**G**) of LNPs with DSPC at different mole ratios. Quantification of mCherry positive cells (**H**) and mean fluorescence intensity (**I**) of HUVECs transfected with DOPE or DSPC containing LNPs encapsulated with mCherry mRNA at 6 h post-transfection. *** *P* < 0.001 versus LNP-2 group. Data are presented as the mean ± SEM

Current FDA-approved LNPs for mRNA vaccines and siRNA delivery utilize DSPC as a helper lipid. However, DSPC was originally designed for siRNA delivery and exhibits low dissociation efficiency for long-chain nucleotides like mRNA [22]. Structurally, DOPE contains a cis-double bond in each aliphatic tail, enhancing mRNA release, whereas DSPC has fully saturated tails that strongly associate with RNA. Mechanistically, DSPC adopts a cylindrical phase, forming stable lipid bilayers, while DOPE adopts a conical shape that transitions to a hexagonal conformation, promoting membrane fusion [23]. To address this limitation, we developed DOPE-containing LNPs, which are more conducive to mRNA release. These LNPs consist of four lipid components: DLin-MC3-DMA, cholesterol, DOPE, and DMG-PEG 2000 (Fig. 1A).

To optimize the lipid composition for mRNA delivery, we synthesized a series of LNPs encapsulated with mCherry mRNA (LNP-1 through LNP-5) with different mole ratios of the four lipids (Fig. S1). Each formulation had a polydispersity index (PDI) of less than 0.2, indicating a monodisperse distribution. Hydrodynamic diameters ranged from 80 to 100 nm (Fig. 1B), and zeta potentials ranged from − 17 to 0 mV (Fig. 1C).

To assess transfection efficiency, HUVECs were treated with LNP1-5 loaded with mCherry mRNA. All formulations significantly enhanced mCherry expression compared to free mRNA delivery (Fig. 1D and E). Notably, LNP-2 achieved the highest transfection efficiency, with 65% of cells being mCherry-positive (Fig. 1D) and a mean fluorescence intensity (MFI) of 240,000 compared to 40,000 in the control group (Fig. 1E). Representative FACS histograms are shown in Fig. S2A. These findings indicate that LNP-2 provides an optimal mole ratio for efficient mRNA delivery.

Given the structural characteristics of DOPE, which supports effective mRNA release, we hypothesized that DOPE-containing LNPs would outperform traditional DSPC-containing LNPs in delivering mRNA. To test this, we synthesized DSPC-containing LNPs (LNP-6 through LNP-8) with mole ratios corresponding to LNP-1, LNP-2, and LNP-5, respectively (Fig. S1). All DSPC-LNPs encapsulated with mCherry mRNA were monodisperse with PDI values < 0.2, had diameters between 95 and 99 nm (Fig. 1F), and exhibited zeta potentials from − 6 to 0 mV (Fig. 1G). Additionally, encapsulation efficiency measured by RiboGreen assays showed that 5 out of the 8 LNP formulations achieved over 80% encapsulation efficiency (Fig. S1).

When comparing transfection efficiency in HUVECs, DOPE-containing LNPs (LNP-1, LNP-2, and LNP-5) consistently showed superior delivery performance relative to their DSPC counterparts (LNP-6, LNP-7, and LNP-8) (Fig. 1H and I). Specifically, LNP-2 showed the highest percentage of mCherry-positive cells (25%) and MFI (73,000), markedly outperforming LNP-7, its DSPC equivalent. Representative FACS histograms are shown in Fig. S2B. These data support that DOPE-containing LNPs are more efficient for mRNA delivery than DSPC-containing LNPs.

Based on these findings, LNP-2 (35:46.5:16:2.5 ratio of DLin-MC3-DMA, cholesterol, DOPE, and DMG-PEG 2000) was selected for further experiments. mRNA-encapsulated LNP-2 formulations were named LNP@mCherry, LNP@Fluc, and LNP@SDF-1α based on the specific mRNA payload (mCherry, firefly luciferase, or SDF-1α, respectively).

### DOPE-LNPs are low cytotoxic, stable, and highly efficient for mRNA delivery in vitro

**Fig. 2:**
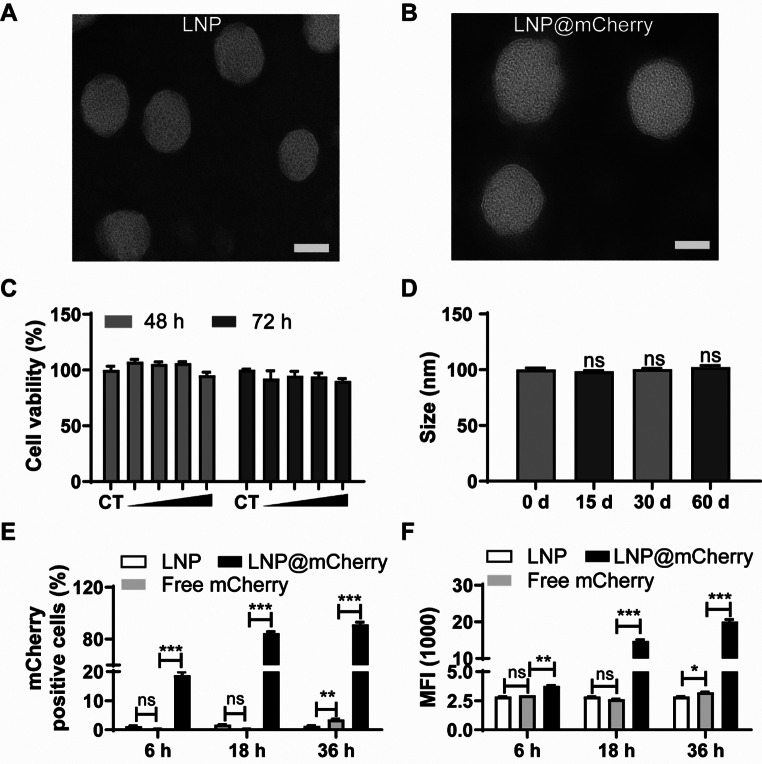
The shape, cytotoxicity, stability of LNPs and their delivery efficiency of mCherry mRNA in ECs at different times. TEM images of empty LNP-2 (**A**) and mcherry mRNA encapsulated LNP-2 (**B**). Scale bar: 50 nm. (**C**) Cytotoxicity was examined by CCK-8 assay of HUVECs incubated with empty LNP-2 at various concentrations for 48–72 h. (**D**) Sizes of LNPs encapsulated with mRNA kept at 4 °C up to 60 days post assembly. Quantification of mCherry positive cells (**E**) and mean fluorescence intensity (**F**) of HUVECs transfected with LNP@mCherry or naked mCherry mRNA for 6, 18, or 36 h. ** *P* < 0.01, *** *P* < 0.001 versus Free mCherry group. Data are presented as the mean ± SEM

Transmission electron microscopy (TEM) images confirmed the spherical morphology of both empty LNP-2 (Fig. 2A) and mCherry mRNA-loaded LNP-2 (Fig. 2B), with diameters consistently around 100 nm. The particles exhibited good dispersion in suspension without notable aggregation.

To evaluate the cytotoxicity of LNP-2, HUVECs were treated with empty LNP-2 at concentrations from 12.5 to 100 µg/mL for both 48 and 72 h. A CCK-8 assay indicated that cell viability was unaffected compared to the control group, suggesting that DOPE-LNPs possess low cytotoxicity (Fig. 2C).

The stability of LNP@mRNA was assessed by monitoring particle size at 4 °C over 15, 30, and 60 days post-assembly. No significant change in particle size (~ 100 nm) was observed, confirming the high stability of these formulations over time (Fig. 2D).

Naked mRNA has been consistently shown to have a limited ability to cross the cell membrane due to its negative charge [24]. To evaluate the mRNA delivery efficiency of LNPs in vitro, we assessed the mCherry fluorescent signal at 6, 18, and 36 h post-transfection of mCherry mRNA. Free mCherry mRNA was used as a control, and LNPs alone served as the vehicle control. Consistent with previous reports, free mCherry mRNA generated a weak fluorescent signal, detectable only at 36 h post-transfection, in contrast to the robust signal observed in the LNP group. At each time points, LNP-2 demonstrated a substantially increased percentage of mCherry-positive cells and higher mean fluorescence intensity (MFI) compared to free mRNA (Fig. 2E and F). Representative FACS histograms are displayed in Fig. S3.

Overall, these results confirm that LNP-2 is non-cytotoxic, highly stable, and can deliver mRNA with high efficiency in vitro.

### LNP@SDF-1α promotes endothelial network formation, proliferation, migration and CXCR4 positive cell adhesion

**Fig. 3:**
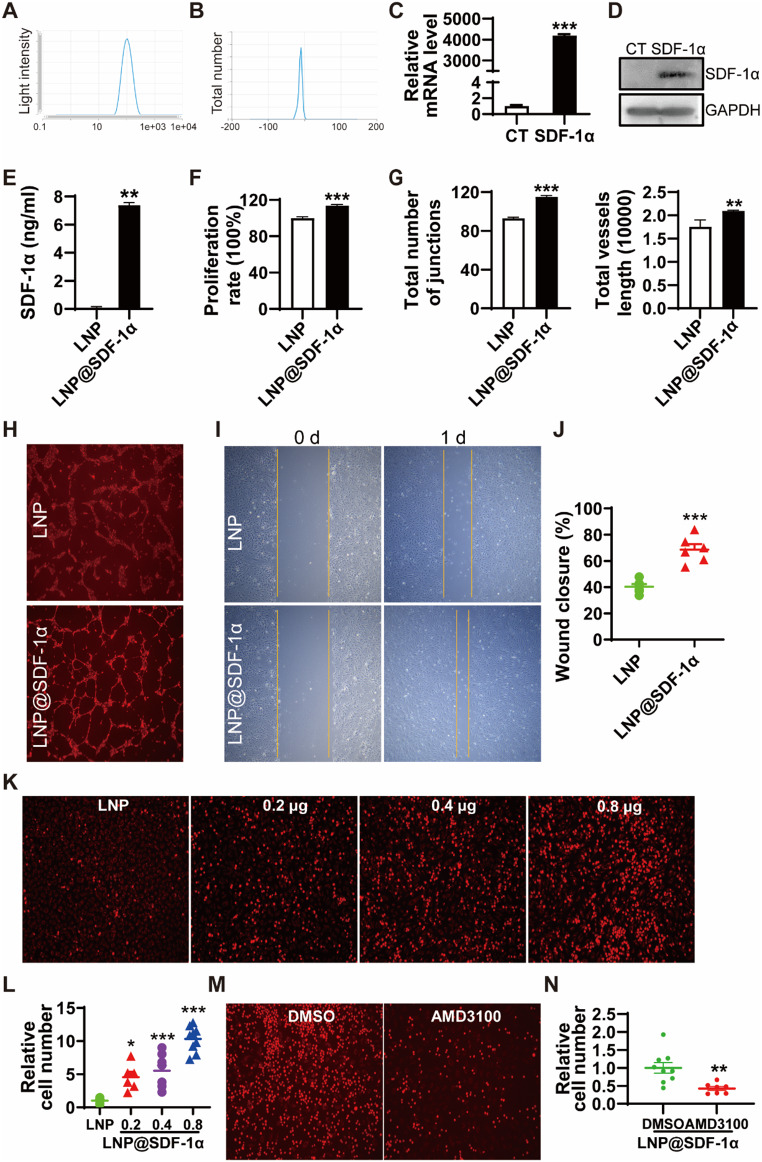
The angiogenesis, migration, and adhesion effects of LNP@SDF-1α in vitro. Sizes (**A**) and zeta potentials (**B**) of LNP@SDF-1α. SDF-1α mRNA was delivered to HUVECs for 24 h. (**C**) SDF-1 mRNA levels. (**D**) Protein levels. (**E**) The secreted SDF-1α protein levels in the culture supernatant examined by ELISA. (**F**) Proliferation of HUVECs. (**G**) Quantification of numbers of junctions and vessel length using AngioTool software. (**H**) Representative images of tube formation of HUVECs treated with LNP or LNP@SDF-1α. (**I**) Representative images of cell migration after scratch. (**J**) Quantification of cell migration in I. (**K**) The adhesion of THP-1 cells to HUVECs. (**L**) Quantification of cell adhesion in K. (**M**) The adhesion of THP-1 cells to HUVECs. HUVECs were treated with 0.4 µg LNP-delivered SDF-1α for 6 h. THP-1 cells were pre-incubated with 10 µM CXCR4 antagonist AMD3100 8HCl for 30 min, then added to ECs. (**N**) Quantification of cell adhesion in M.* *P* < 0.05, ** *P* < 0.01, *** *P* < 0.001. Data are presented as the mean ± SEM

The hydrodynamic diameter of LNP@SDF-1α was measured at 110 nm (Fig. 3A), with a zeta potential of -10 mV (Fig. 3B). To evaluate the delivery efficiency of SDF-1α mRNA, HUVECs were transfected with LNP@SDF-1α for 24 h, resulting in significantly increased mRNA levels (Fig. 3C), protein expression (Fig. 3D) and secretion into culture supernatant (Fig. 3E) as assessed by RT-PCR, western blot, and ELISA assay compared to the control.

To compare the expression efficiency of mRNA and plasmid, the SDF-1α level in the supernatant was tested by ELISA. The SDF-1α protein level expressed by mRNA was significantly higher than that of plasmid (Fig. S4).

The effect of LNP@SDF-1α on the survival of HUVECs was examined by CCK-8 kit. As shown in Fig. 3F, LNP@SDF-1α could slightly promote the proliferation of HUVECs. To assess angiogenic potential, HUVECs were transfected with LNP@SDF-1α or control LNP for 6 h before being subjected to a Matrigel tube formation assay. As shown in Fig. 3G and H, LNP@SDF-1α-treated cells displayed a marked increase in tube formation compared to controls. Quantitative analysis using AngioTool software confirmed significant increases in the number of junctions and total vessel length (Fig. 3G) in the LNP@SDF-1α group.

The migration capacity of HUVECs post-transfection with LNP@SDF-1α was evaluated by scratch assay. LNP@SDF-1α treatment significantly enhanced migration, with wound closure increasing from 42.3 to 60.9% (Fig. 3I and J).

Additionally, the effect of LNP@SDF-1α on the adhesion of human CXCR4-positive THP-1 cells to ECs was investigated. LNP@SDF-1α-treated HUVECs demonstrated enhanced monocytic cell adhesion (Fig. 3K), which was further increased in a dose-dependent manner with escalating concentrations of SDF-1α mRNA (Fig. 3L). To further determine whether the SDF-1α-induced enhancement is dependent on its receptor CXCR4, we applied CXCR4 antagonist AMD3100 8HCl and observed a significant reduction in cell adhesion (Fig. 3M and N). This results confirm that SDF-1α-mediated promotion of cell adhesion is CXCR4-dependent.

### LNP@SDF-1α enhances angiogenesis in the matrigel plug assay

**Fig. 4:**
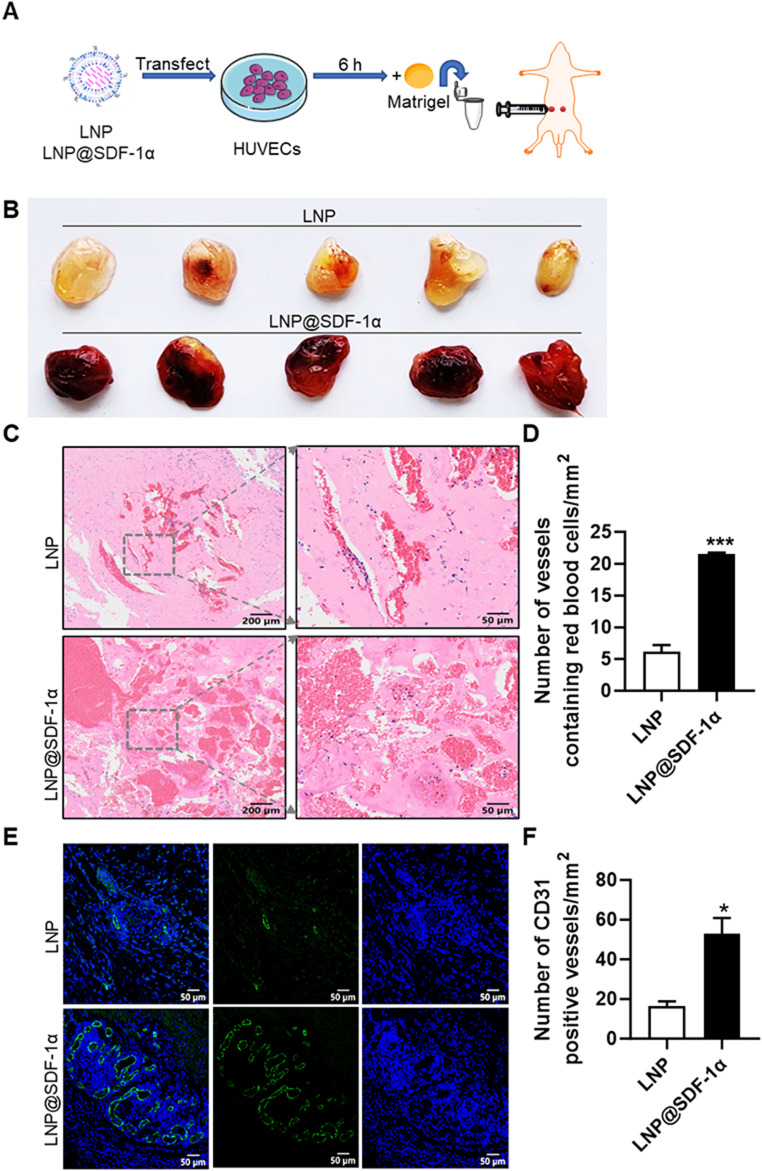
The angiogenesis effects of LNP@SDF-1α in vivo. (**A**) Schematic of the Matrigel plugs assays. (**B**) Bright-field image of LNP@SDF-1α induced angiogenesis in Matrigel plugs. (**C**) Representative images of hematoxylin and eosin staining of Matrigel plugs with the indicated treatment. (**D**) Quantification of blood vessels containing red blood cells. (**E**) Immunofluorescent staining of Matrigel plugs. (**F**) Quantification of the number of vessels in **E**. * *P* < 0.05, *** *P* < 0.001. Data are presented as the mean ± SEM

To assess the angiogenic potential of LNP@SDF-1α in vivo, we utilized a mouse Matrigel plug assay (Fig. 4A).

HUVECs treated with LNP@SDF-1α were embedded in Matrigel, while LNP-treated cells served as controls. These Matrigel plugs were then injected subcutaneously into 8-week-old C57BL/6 mice, and harvested five days post-injection for analysis. Matrigel plugs containing HUVECs transfected with SDF-1α mRNA displayed a darker red color, indicative of increased blood content and vascular density compared to control plugs (Fig. 4B).

HE staining revealed a significant increase in the number and size of blood vessels containing red blood cells in LNP@SDF-1α-treated HUVEC plugs compared to the control group (Fig. 4C and D). Immunofluorescent staining for CD31 further confirmed enhanced vascularization in the LNP@SDF-1α-treated group (Fig. 4E and F).

These results conclusively demonstrate that LNP@SDF-1α promotes angiogenesis in vivo, supporting its potential as an effective therapeutic for vascular regeneration.

### Intramuscular injection of LNP@SDF-1α enhanced the angiogenesis, blood perfusion, and preserved limb function in murine hindlimb ischemia model

**Fig. 5:**
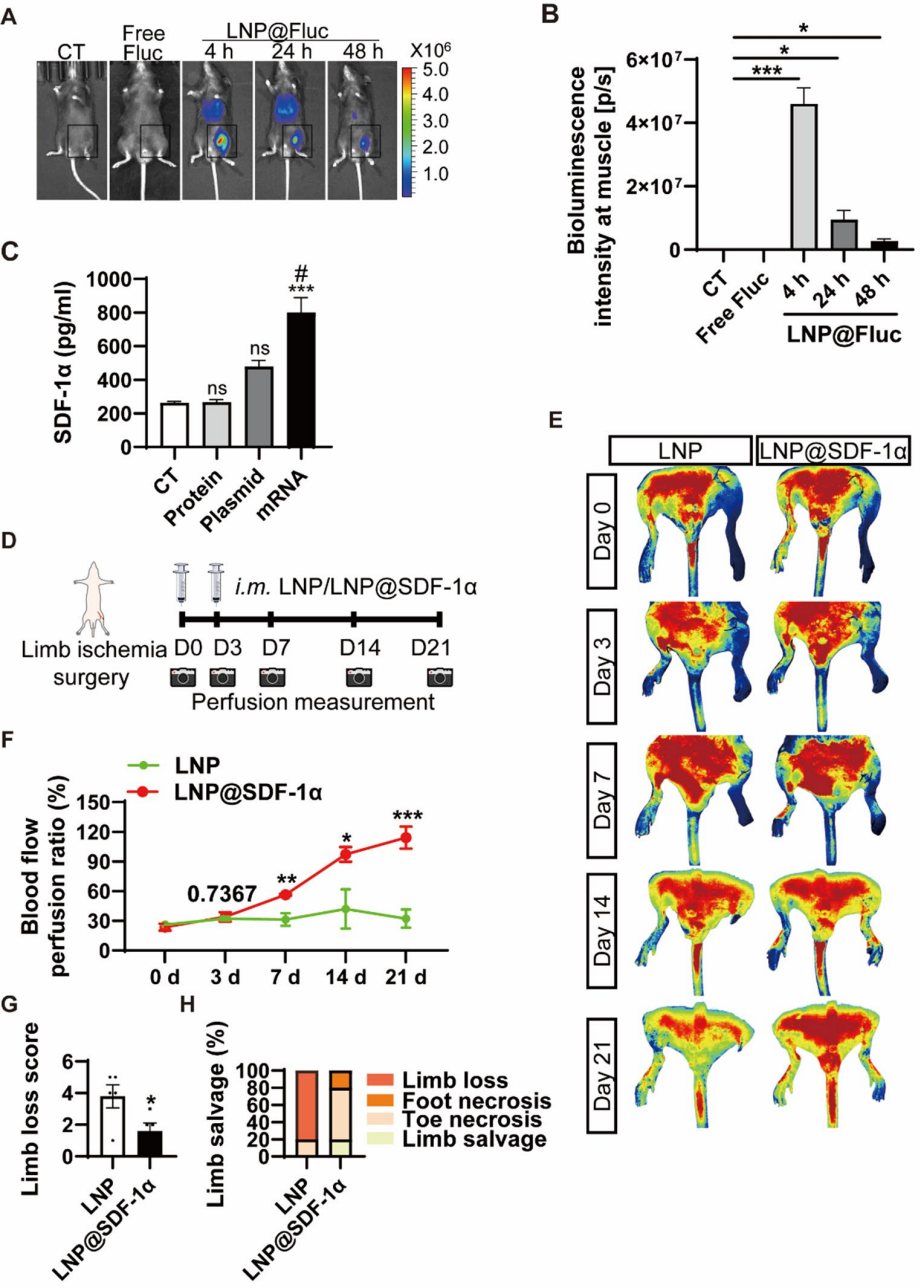
Therapeutic effects of LNP@SDF-1α in the lower limb ischemia model at 21 d. (**A**) The expression of Free Fluc mRNA or Fluc mRNA delivered by LNP in vivo. (**B**) Quantification of fluorescence intensity in A. (**C**) The SDF-1α protein level in the plasma from mice treated with SDF-1α protein, plasmid or mRNA. * versus the control group; # versus the plasmid group. (**D**) Schematic of the treatment of the lower limb ischemia. (**E**) Laser Speckle Contrast Imaging of the ischemic hindlimb on Days 0, 3, 7, 14, and 21 after injury. (**F**) Quantification of the blood flow perfusion ratio of the ischemic limb compared with the nonischemic limb (*n* = 5). (**G**) Limb lost score in ischemic limb. (**H**) Scoring for the physiological status of ischemic limbs at Day 21. * *P* < 0.05, ** *P* < 0.01, *** *P* < 0.001. # *P* < 0.05. Data are presented as the mean ± SEM

To evaluate the local expression dynamics of LNP-mediated mRNA delivery in vivo, we administered an intramuscular injection of LNP encapsulating 4 µg of Fluc mRNA into mice and monitored whole-body bioluminescence at 4, 24, and 48 h post-injection. While 4 µg of naked Fluc mRNA was also examined to determine their delivery efficiency. Bioluminescence served as an indicator of Fluc expression. At 4 h post-injection (Fig. 5A and B), we only observed robust bioluminescence at the local injection site treated with LNP encapsulated Fluc mRNA, indicating that LNP successfully delivered mRNA, which was rapidly translated into functional protein in vivo. Bioluminescence was also detectable at 24 and 48 h post-injection, suggesting a sustained expression for at least two days. Free Fluc mRNA without LNP exhibited minimal bioluminescence, similar to LNP group.

To directly compare the delivery efficiency of recombinant protein, plasmid, and mRNA forms of SDF-1α, we injected SDF-1α protein, naked SDF-1α plasmid, and SDF-1α mRNA encapsulated by LNP into the gastrocnemius. After 4 h, the plasma was collected, and SDF-1α protein levels were measured by ELISA. In both the protein and plasmid injection groups, SDF-1α levels remained comparable to those in untreated control plasma (Fig. 5C). This aligns with previous reports indicating that recombinant proteins are rapidly degraded in tissues, while plasmid-driven expression in vivo is delayed and relatively low. In contrast, SDF-1α mRNA administration resulted in robust protein expression, highlighting its potential for rapid and efficient therapeutic delivery.

We next assessed the therapeutic efficacy of LNP@SDF-1α on revascularization in a mouse hindlimb ischemia model (Fig. 5D). LNP@SDF-1α or control LNP was injected intramuscularly immediately after surgery and again three days later. Laser Speckle Contrast Imaging was used to monitor tissue reperfusion (Fig. 5E), showing significantly faster blood flow recovery in ischemic limbs treated with LNP@SDF-1α compared to controls, particularly at 7, 14, and 21 days post-surgery (Fig. 5F). The limb loss score and quantitative scores of limb salvage (Fig. 5G and H) were also markedly improved in the LNP@SDF-1α group. A higher Limb loss score indicates more adverse symptoms. These findings suggest that injection of LNP@SDF-1α can improve blood perfusion and help preserve limb function in the ischemic limbs of mice.

**Fig. 6:**
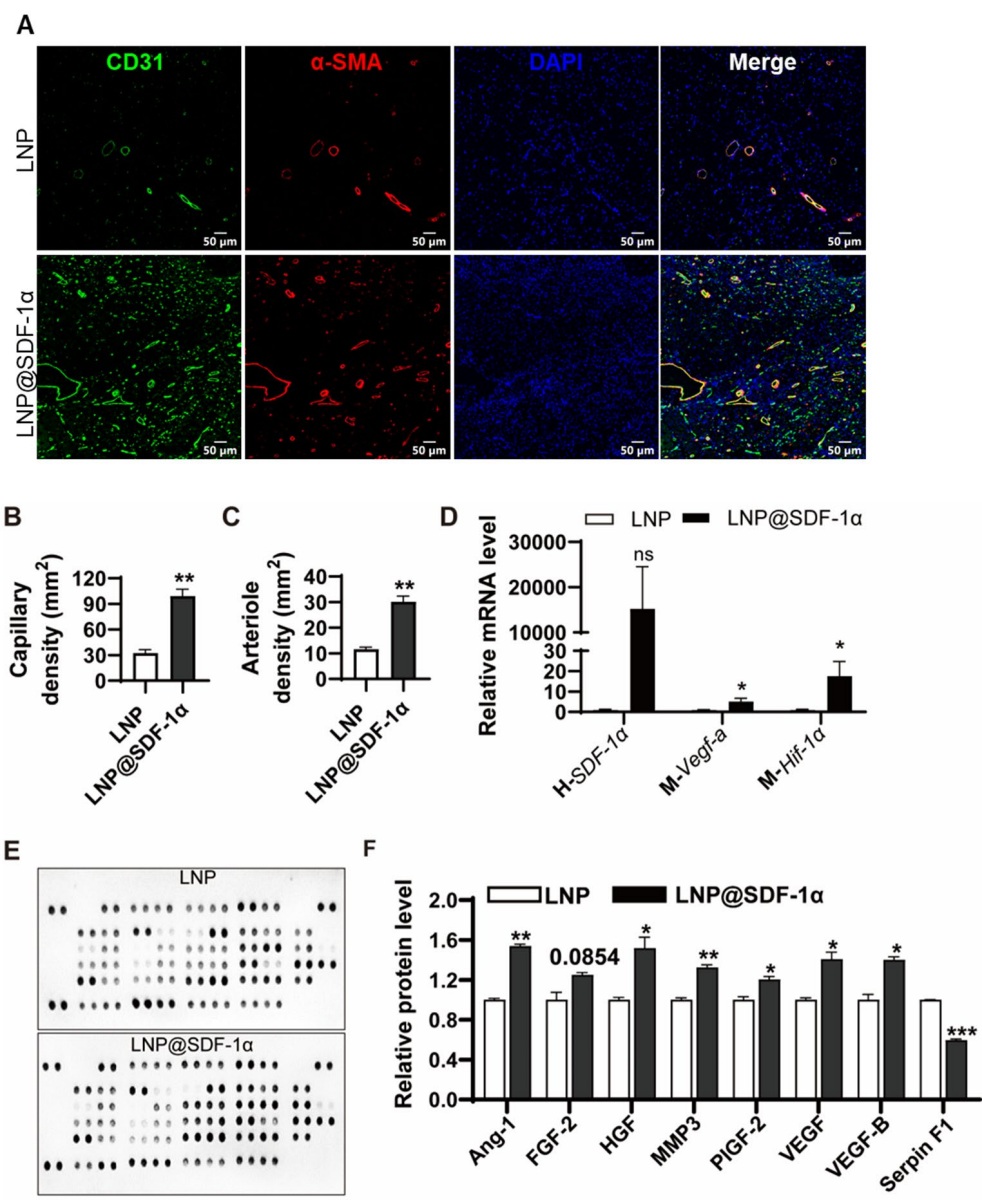
LNP@SDF1α significantly promote angiogenesis at Day 21 following hindlimb ischemia. (**A**) Representative images of immunofluorescent staining with α-SMA (red), CD31 (green), and DAPI for nuclei (blue) on Day 21 after surgery. Quantification of the capillary (**B**) and artery density (**C**) in A. (**D**) The relative mRNA levels of human *SDF-1α*, mouse *Vegf-a*, and *Hif-1α* in muscles tested by RT-PCR. (**E**) The mouse angiogenesis array detected multiple analytes in muscles. (**F**) Quantification of selected angiogenesis-related proteins by gray analysis. * *P* < 0.05, ** *P* < 0.01, *** *P* < 0.001. Data are presented as the mean ± SEM

To examine angiogenesis, immunofluorescence staining was performed on gastrocnemius muscles at 21 days post-surgery. Capillary (vessels contain CD31^+^ EC layer) density and arteriole (vessels contain CD31^+^ EC layer and α-SMA^+^ smooth muscle layer) density were significantly elevated in the LNP@SDF-1α treated group compared to controls (Fig. 6A). Quantitative analysis further confirmed that LNP@SDF-1α significantly increased the number of capillaries (Fig. 6B) and arterioles (Fig. 6C).

Additionally, LNP@SDF-1α treatment upregulated *Vegf* and *Hif-1α* mRNA levels, and notably, human *SDF-1α* mRNA was still detectable at 21 days post-injection (Fig. 6D).

We also examined angiogenesis-related protein expression using an angiogenesis array (Fig. 6E and F). LNP@SDF-1α treatment elevated pro-angiogenic factors such as angiopoietin-1 (Ang-1), hepatocyte growth factor (HGF), placental growth factor-2 (PIGF-2), and VEGF, along with an increase in matrix metalloproteinase-3 (MMP-3) levels. In contrast, the anti-angiogenic factor serpin F1 was significantly downregulated.

Histological examination of major organs (liver, spleen, and kidney) revealed no significant toxicity associated with LNP or LNP loaded with mRNA, supporting the favorable safety profile of our newly developed DOPE-LNP and modified mRNA (Fig. S5).

## Discussion

In this study, we demonstrate that SDF-1α mRNA therapy significantly enhances vascular regeneration in a murine model of hindlimb ischemia, a condition that mimics human PAD. Our findings provide compelling evidence that localized delivery of SDF-1 mRNA restores vascular density and improves blood perfusion, leading to enhanced functional recovery in ischemic tissues. These results highlight the therapeutic potential of SDF-1α mRNA and suggest its broader applicability for ischemic disease treatment.

SDF-1α has long been recognized for its critical role in vascular regeneration, particularly through its ability to mobilize EPCs and direct them to sites of ischemic injury sites [8, 9, 12]. SDF-1 can also attract monocytes that produce cytokines and metalloproteinases that facilitate arteriogenesis [25]. In addition, it plays a pivotal role in recruiting bone marrow-derived mononuclear cells (BM-MNC), which have shown benefits in improving PAD outcomes by clinical trial results [26–28]. For example, a meta-analysis of 10 randomized, placebo‐controlled trials (499 patients) showed that BM-MNC therapy improves ankle-brachial index, resting pain, and pain‐free walking time [26]. Another meta‐analysis of 19 randomized, placebo‐controlled trials (837 patients) found that cell therapy modestly reduced amputation risk and improved amputation-free survival and wound healing [28]. Beyond vascular regeneration, SDF-1 also recruits CXCR4^+^ satellite cells and myoblasts, which contribute to muscle repair [29, 30]. Consistent with our findings, a recent study has reported mesenchymal stem cell-derived SDF-1α can restore vascular regeneration in the murine hindlimb ischemia model [21].

However, previous clinical applications of SDF-1-based therapies have faced significant challenges, particularly in terms of stability and delivery *in vivo.* A phase 2B clinical trial involving 109 PAD patients with critical limb ischemia found no significant improvement in outcomes with intramuscular plasmid encoding SDF-1 in conjunction with endovascular surgery [15]. The limited efficacy of SDF-1 protein therapies has been largely attributed to their short half-life and rapid degradation in vivo.

Our work addresses these limitations by employing mRNA-based SDF-1α delivery to enable quick, robust, and localized expression in ischemic tissues using a newly developed LNP delivery system. Unlike current FDA-approved LNPs, which contain DSPC-a helper lipid originally optimized for siRNA delivery with low dissociation efficiency for long-chain nucleotides like mRNA-we developed a DOPE-containing LNP formulation to enhance mRNA delivery. Structurally, DOPE features a cis-double bond in each aliphatic tail, facilitating mRNA release, while its conical shape promotes membrane fusion by transitioning to a hexagonal conformation [22, 23]. Indeed, replacing DSPC with DOPE significantly improved mRNA delivery efficiency.

SDF-1α mRNA therapy markedly increased capillary density in treated animals compared to controls, consistent with SDF-1α’s established role in endothelial migration and tube formation. Importantly, SDF-1α mRNA therapy not only promoted angiogenesis but also facilitated arteriogenesis, as evidenced by the increased density of arterioles. This dual effect is particularly relevant for PAD, which involves not only microvascular perfusion deficits but also impaired arterial conduit formation. By facilitating both angiogenesis and arteriogenesis, SDF-1α mRNA therapy addresses the multifaceted vascular impairments characteristic of PAD.

Our findings also indicate that SDF-1α mRNA therapy enhances tissue perfusion, likely as a result of increased microvascular and arteriolar density. These improvements in perfusion are critical for effective tissue repair and regeneration in ischemic conditions. Despite these promising results, several questions remain. For instance, the long-term effects of SDF-1 mRNA therapy on vascular stability and functionality need further investigation to ensure the durability and effectiveness of the newly formed vessels in supporting tissue perfusion. Additionally, the therapeutic efficacy of SDF-1α mRNA in pathological conditions such as hyperglycemia or hyperlipidemia remains to be explored.

In conclusion, our study underscores the potential of SDF-1α mRNA therapy as a powerful approach for vascular regeneration, capable of restoring vascular density and improving blood perfusion in ischemic tissues. This strategy holds promise for PAD treatment, especially for patients with critical limb ischemia who have limited therapeutic options. By leveraging the unique attributes of mRNA-based therapies, we have successfully overcome the challenges associated with protein delivery and established a novel strategy for vascular regeneration in ischemic disease.

## Electronic supplementary material

Below is the link to the electronic supplementary material.

Supplementary Material 1

## Data Availability

No datasets were generated or analysed during the current study.
