# Supplementary material for: SDF-1α mRNA therapy in peripheral artery disease
Source: Angiogenesis. 2025 May 2;28(3):26. doi: 10.1007/s10456-025-09979-3 (PMC12048462; doi:10.1007/s10456-025-09979-3)

**Figure legends**

Figure S1. The composition and the encapsulation efficiency of different particles.

Figure S2. mCherry mRNA delivery efficiency of LNP. HUVECs were treated with different samples for 24 h (A) and 6 h (B). The cells were collected for flow cytometry. Representative images of cellular uptake.

Figure S3. mCherry mRNA delivery efficiency of LNP. HUVECs were treated with different samples for indicated times (6, 18, and 36 h). The cells were collected for flow cytometry. Representative images of cellular uptake.

Figure S4. The SDF-1α protein level in the supernatant of cells transfected with SDF-1α mRNA or plasmid tested by ELISA.* versus the control group, # versus the plasmid group. ** *P* < 0.01, *** *P* < 0.001, ## *P*< 0.01. Data are presented as the mean ± SEM.

Figure S5. Histopathological studies of major tissues (live, kidney, and spleen) obtained from mice injected with various nanoparticles.


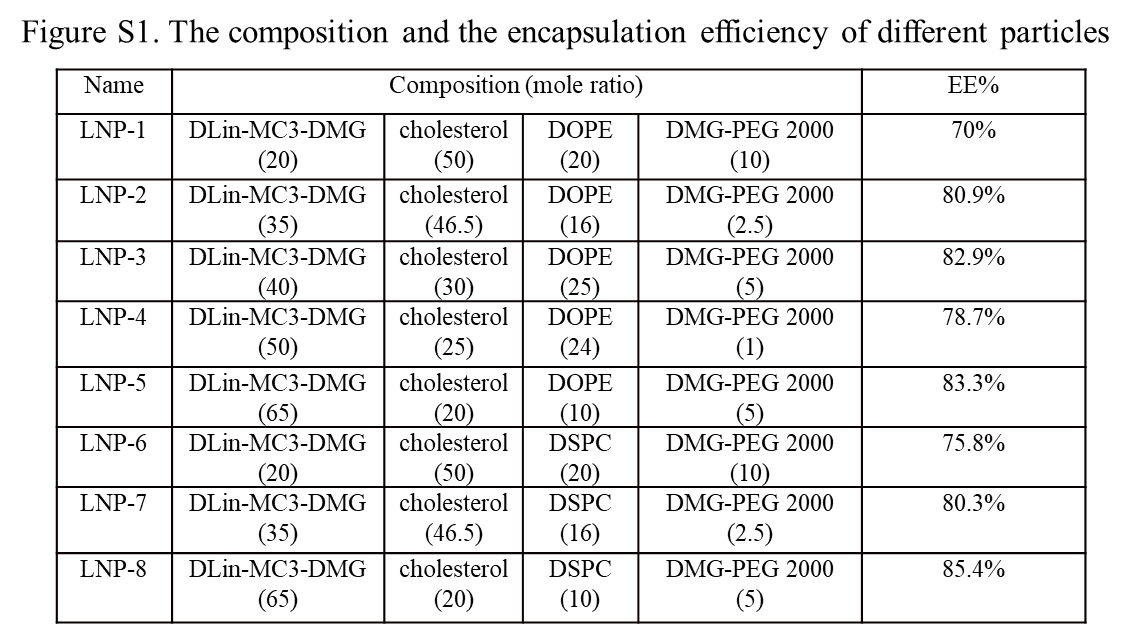


Figure S2


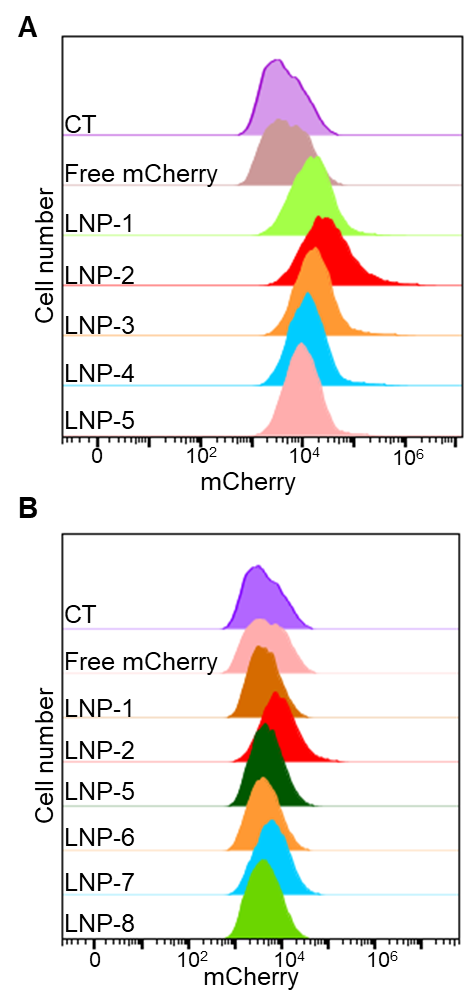


Figure S3


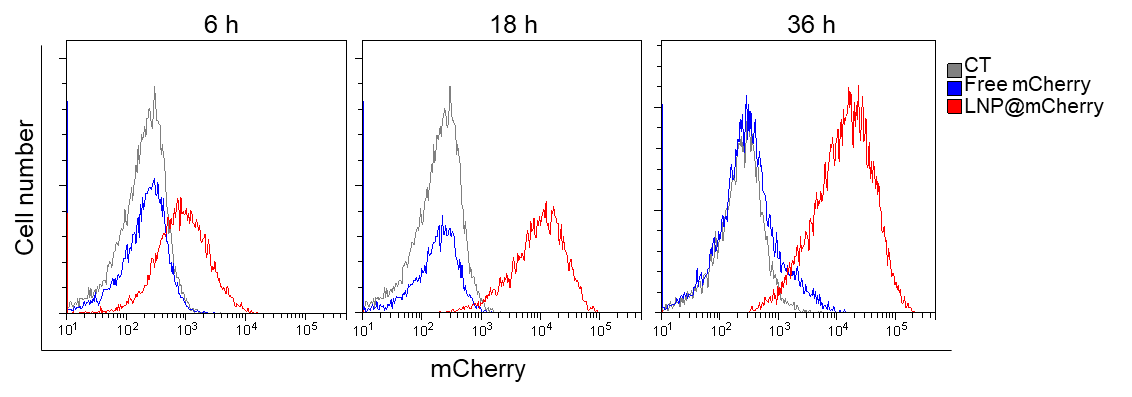


Figure S4

Figure S5


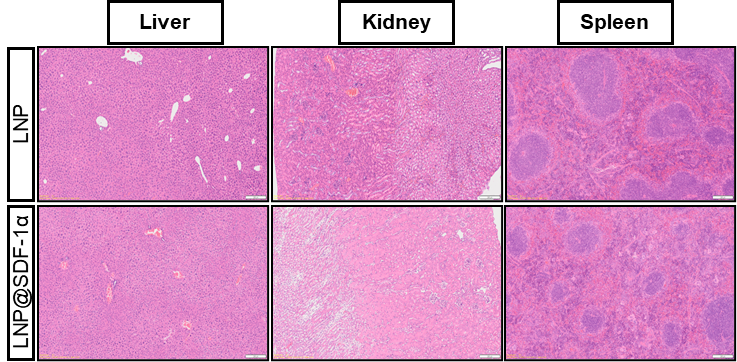

Supplement: Supplementary file 1 — Supplementary Material 1 [file 10456_2025_9979_MOESM1_ESM.docx]
